# Management of perioperative anticoagulation in patients with mechanical heart valve replacement undergoing laparoscopic cholecystectomy: a case report of postoperative cerebral embolism

**DOI:** 10.3389/fsurg.2024.1404436

**Published:** 2024-08-21

**Authors:** Wei He, Panli Tang

**Affiliations:** ^1^ Department of Ophthalmology, Sichuan Taikang Hospital, Chengdu, China; ^2^ Department of Cardiothoracic Surgery, Sichuan Taikang Hospital, Chengdu, China

**Keywords:** perioperative anticoagulation, mechanical heart valve replacement, abdominal surgery, postoperative cerebral embolism, warfarin bridging therapy

## Abstract

Patients with mechanical heart valve replacement require lifelong anticoagulation therapy, and additional surgeries can pose a risk of bleeding and thromboembolic events due to the need for perioperative anticoagulation management. Here, we present a case report of a patient who underwent laparoscopic cholecystectomy (LC) and endoscopic retrograde cholangiopancreatography (ERCP) after mechanical heart valve replacement and experienced postoperative cerebral embolism. The management of perioperative anticoagulation in these patients is discussed, including strategies for minimizing the risks of bleeding and thromboembolic events during and after surgery.

## 1 Introduction

Mechanical heart valve replacement surgery remains a crucial intervention for patients with valvular heart diseases, necessitating lifelong anticoagulation therapy to prevent thromboembolic events (1). However, when these patients require additional surgeries, particularly abdominal procedures, the management of perioperative anticoagulation becomes challenging due to the delicate balance required to mitigate both bleeding and thromboembolic risks (2). Despite the availability of guidelines from national organizations and international societies, real-world scenarios often present complexities that are not fully addressed by these guidelines. Herein, we present a case report highlighting the complexities of managing perioperative anticoagulation in a patient with a history of mechanical heart valve replacement who underwent laparoscopic cholecystectomy and subsequently experienced postoperative cerebral embolism. The motivation for reporting this case is threefold: (1) to illustrate the practical challenges and clinical decision-making nuances in managing such high-risk patients, (2) to underscore the importance of meticulous perioperative monitoring and individualized patient management, and (3) to share insights and lessons learned that may contribute to improved outcomes and inform future clinical practice. By detailing this case, we aim to provide valuable information for clinicians facing similar situations and to emphasize the need for interdisciplinary collaboration in managing complex cases involving anticoagulation therapy.

## 2 Case presentation

**Figure 1:**
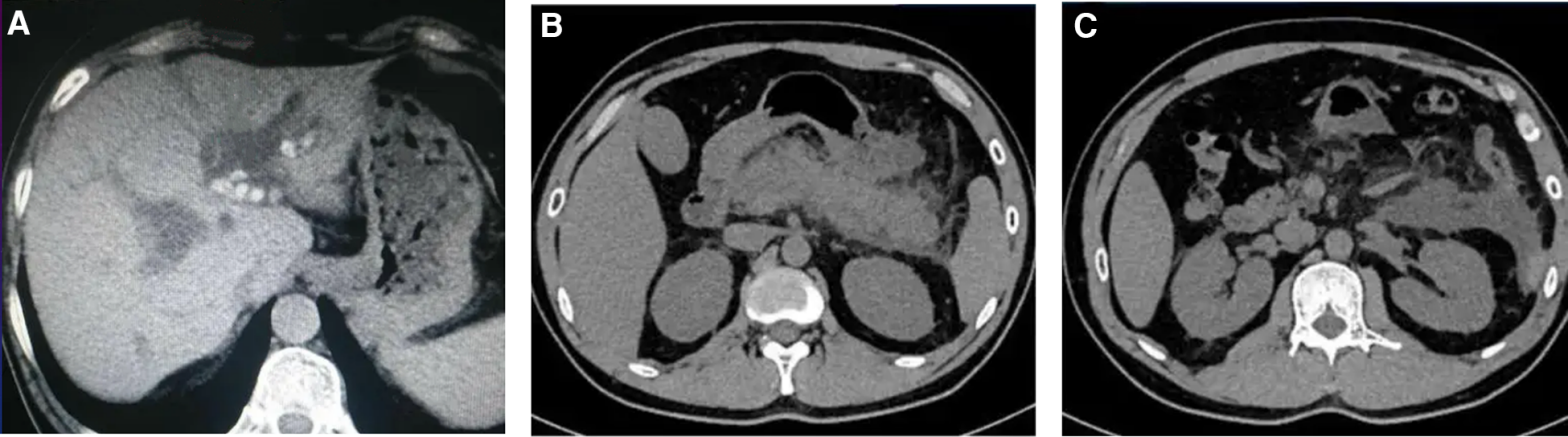
Urgent abdominal computed tomography (CT) scan upon admission showed multiple gallstones in the gallbladder and acute pancreatitis. **(A)** Multiple gallstones in the gallbladder; **(B,C)** The swelling of the pancreas with peri-pancreatic exudation and liquid collection.

The patient is a 53-year-old female who was admitted to the emergency department due to upper abdominal pain for two days. The physical examination showed there was no jaundice of the skin and sclera, the abdomen was soft and flat, there was tenderness in the left and right upper abdomen, but no muscle tension or rebound pain, and Murphy's sign was positive. An urgent abdominal computed tomography (CT) scan upon admission showed acute pancreatitis, multiple gallstones in the gallbladder, and a high possibility of a stone at the lower end of the common bile duct (see Figure 1). The 12-lead electrocardiogram confirmed atrial fibrillation and the heart rate was about 80 beats per minute. An urgent amylase test was performed and the result was 3,573 U/L. Table 1 showed the major laboratory values of the patient. Therefore, the patient was admitted to our hospital with a diagnosis of “acute pancreatitis and cholecystitis with gallstones” and was treated accordingly. The patient had a history of rheumatic mitral stenosis and underwent mechanical mitral valve replacement surgery 20 years ago. She has been taking warfarin since the surgery and has not experienced any thrombosis or bleeding. Beyond this, her personal medical history showed no other problems.

**Table 1:** Major laboratory values of the patient.

	Reference range	The day of admission	The 16th day after admission (the 2nd day before operation)	The 2nd day after operation	The 5th day after operation
WBC(×10^9^/L)	4.0–10.0	12.53	5.32	11.98	9.97
NEUT(%)	45–77	88.5	71.4	83.32	79.8
CRP(mg/L)	≤5.0	23.21	3.24	12.24	3.8
TBIL(μmol/L)	5.1–19.0	25.88	16.88	21.15	14.34
DBIL(μmol/L)	1.7–6.8	13.9	3.2	9.8	7.6
ALT(U/L)	<41	15.32	9.9	67.8	45.1
AST(U/L)	<40	9.8	8.8	55.3	37.9
ALP(U/L)	40–150	146.55	126.75	166.65	58.8
GGT(U/L)	11–50	235.52	66.53	88.43	49.3
TBA(μmol/L)	<10	12.12	9.2	10.11	8.8
Blood amylase(U/L)	25–135	3,573	99	121	94
PT(s)	10.0–14.0	28.11	12.5	14.8	17.6
INR	0.8–1.5	2.6	1.12	1.17	1.45

WBC, white blood cell count; NEUT, neutrophil; CRP, C-reactive protein; TBIL, total bilirubin; DBIL, direct bilirubin; ALT, alanine transaminase; AST, aspartate aminotransferase; ALP, alkaline phosphatase; GGT, glutamyl transpeptidase; TBA, total bile acid; PT, prothrombin time; INR, International Normalized Ratio.

**Figure 2:**
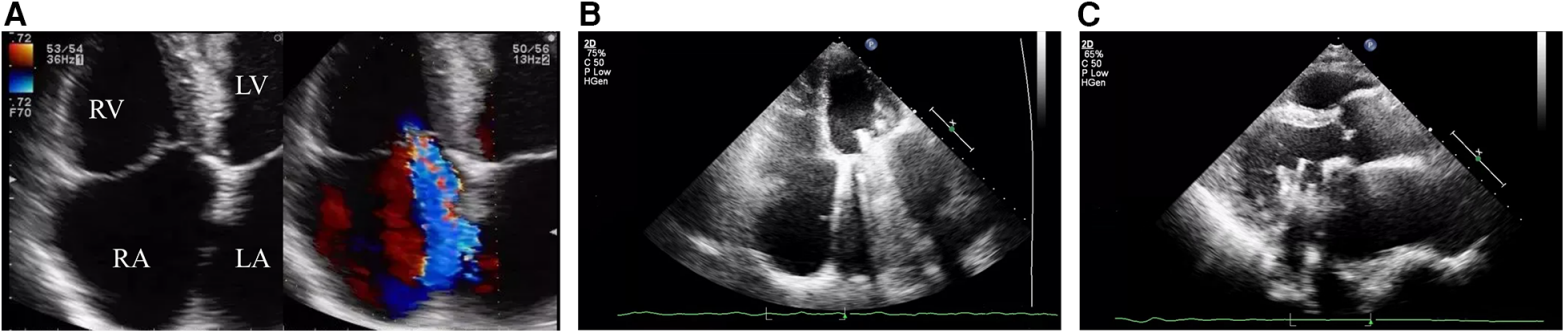
The echocardiogram after the patient was admitted showed mild tricuspid regurgitation with no other significant abnormalities and the mechanical mitral valve functioned normally. **(A)** The apical four-chamber view showed mild tricuspid regurgitation; **(B)** The apical four-chamber view shows strong echogenicity of the mechanical mitral valve prosthesis with large “comet tail sign”; **(C)** Left ventricular long-axis view.

**Figure 3:**
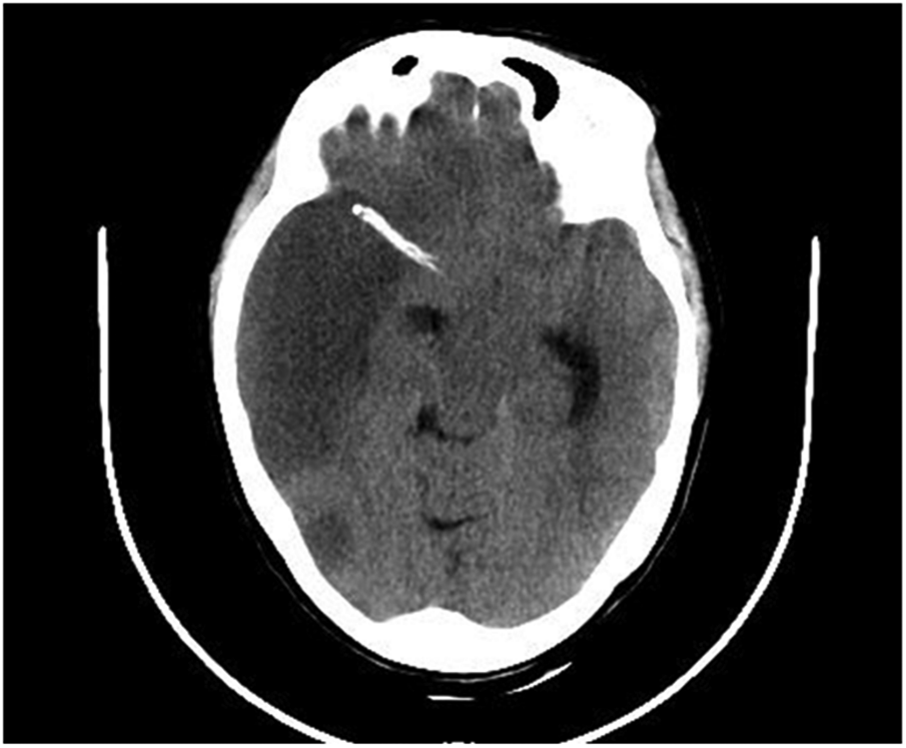
Follow-up cranial CT scan after stent implantation revealed hyperdensity in the right middle cerebral artery territory, indicating changes related to the stent implantation, and an infarction in the right frontal, temporal, parietal, and occipital lobes with brain edema was identified.

During this hospitalization, the patient's diagnosis was relatively clear, and biliary-related acute pancreatitis was considered the most likely cause. Therefore, the treatment plan was to control the symptoms of pancreatitis first, and then performed a LC and ERCP to achieve a radical cure. On the second day of hospitalization, routine coagulation tests revealed a prothrombin time (PT) of 29.80 s and an international normalized ratio (INR) of 2.69. Considering the safety of the surgery, warfarin was discontinued and replaced with subcutaneous injection of 4100AXa units of low molecular weight heparin (LMWH) calcium every 12 h, which was used until the day before the surgery, totaling 18 days. Before the surgery, an echocardiogram showed that the patient had mild tricuspid regurgitation and mild pulmonary hypertension after mechanical valve replacement of the mitral valve (see Figure 2). There were no significant abnormalities found in cervical vascular color doppler ultrasound and electrocardiogram, etc. Before the surgery, the patient received anti-inflammatory drugs, acid-inhibiting therapy and intravenous rehydration treatment. The surgery was performed on after the symptoms of pancreatitis improved significantly. The ERCP procedure revealed a stone at the lower end of the common bile duct, which was successfully removed. The bile duct was subsequently checked for any residual stones, and none were found. The procedure also confirmed choledocholithiasis as initially suggested by the CT scan. During the LC, multiple gallstones were observed in the gallbladder. The gallbladder was inflamed and thickened, consistent with cholecystitis. The surgery was performed smoothly with approximately 10 ml of intraoperative bleeding. On the first day after the surgery, warfarin 2.5 mg was given orally to the patient at night. On the second and fifth days after the surgery, routine coagulation tests were reassessed, and the results showed PT of 14.80 s and 17.60 s and INR of 1.17 and 1.45, respectively. On the seventh day after the surgery, the patient experienced dizziness and fell to the ground while using the toilet. She had unclear speech, left limb weakness, and was immediately given electrocardiographic monitoring and oxygen inhalation. Considering the patient's symptoms and signs, cardiovascular and cerebrovascular diseases were suspected. Urgent cranial CT was performed, and a consultation with the department of neurology was requested simultaneously. The cranial CT showed slightly reduced density in the right frontal, temporal, and parietal lobes compared to the contralateral side. After consulting the department of neurology, occlusive cerebrovascular disease was considered, and given the patient's medical history, the possibility of a cardiac embolism was high. Therefore, digital subtraction angiography (DSA) examination and catheter-directed thrombectomy were recommended. On the second day after the surgery, cranial CT was reexamined, which showed a high-density shadow in the right middle cerebral artery running area, considered to be related to stent implantation. Additionally, there was an infarct in the right frontal, temporal, and parietal lobes and brain swelling (see Figure 3). The patient stayed in the ICU for 7 days, and her condition improved before she was discharged.

## 3 Discussion

Patients who have undergone mechanical valve replacement surgery need to receive lifelong anticoagulant therapy (3). If these patients require surgery again in the future, doctors must not only pay attention to the risk of bleeding during and after the surgery, but also consider the risk of thromboembolism brought about by perioperative anticoagulant prophylaxis (4). Of course, for minor procedures such as tooth extraction, breast lump excision, endoscopy, etc., anticoagulant therapy generally does not need to be stopped. However, for major elective operations, it is necessary to eliminate the anticoagulant effect in the body for a period of time before and after the operation. Managing anticoagulation in patients with mechanical heart valves undergoing non-cardiac surgery is complex and requires a balance between reducing the risk of thromboembolism and minimizing bleeding complications. Key strategies include preoperative risk assessment to evaluate thromboembolic and bleeding risks, discontinuation of warfarin approximately 5 days before surgery, and initiation of bridging therapy with LMWH or unfractionated heparin (UFH) (5). Intraoperative anticoagulation monitoring, such as activated clotting time (ACT), may be considered for high-risk patients. Postoperatively, warfarin should be resumed 12–24 h after surgery, with bridging therapy continued until the INR is within the therapeutic range for at least 24–48 h (6). Frequent monitoring of coagulation profiles, including daily anti-Xa levels in patients receiving LMWH, is crucial to ensure adequate anticoagulation. However, there is no consensus on the safe duration of stopping anticoagulant therapy for non-cardiac surgeries currently, and different regions and hospitals have developed more than 10 schemes for stopping anticoagulant therapy for patients with mechanical valve undergoing non-cardiac surgery (7). Some scholars believe that it is relatively safe to stop anticoagulant therapy for about one week due to excessive anticoagulation or the need for non-cardiac surgeries (8), but this puts the patient at a high risk of potential thrombosis (9).

Perioperative stroke refers to ischemic or hemorrhagic cerebrovascular accidents that occur during or within 3–30 days after surgery (10). Generally, these strokes are mainly ischemic or thrombotic rather than hemorrhagic (11). Among this, emboli most commonly originate from the heart, as the altered hemodynamics, perioperative hypercoagulable state, and preventative anticoagulation measures make the diseased or prosthetic heart valves the most likely location for thrombus formation (12). The American College of Chest Physicians (ACCP) guidelines recommend that oral anticoagulants should be discontinued approximately 5 days before major surgery to allow the INR to return to normal range, and LMWH should be started as a “bridge therapy.” The dose and frequency of LMWH injections depend on the individual patient's risk of thrombosis (13). Oral warfarin should be started 12–24 h after surgery because it takes approximately 3–5 days for warfarin to have an antithrombotic effect. Therefore, warfarin and LMWH should be used in combination postoperatively until the INR reaches therapeutic range and remains stable for 1–2 days before discontinuing LMWH. The patient had two risk factors for brain stroke: atrial fibrillation (AF) and a mechanical heart valve. AF is a well-known significant risk factor for brain stroke (14), and it is essential to perform a cardiac ultrasound before surgery in patients with atrial fibrillation to rule out left atrial thrombi. In this case, since the patient's electrocardiogram monitoring before and after surgery showed stable atrial fibrillation and preoperative cardiac ultrasound did not detect any thrombi, we believe that the cerebral embolism was more likely to have originated from the mechanical heart valve. In addition, postoperative coagulation monitoring showed a low INR, indicating insufficient anticoagulation, which increased the risk of thrombus formation and embolism at the site of the mechanical valve (15). Although we cannot completely rule out atrial fibrillation as a source of the embolism, considering the patient's medical history and postoperative clinical manifestations, we believe that the mechanical heart valve is the more likely source of the embolism. In addition, although the patient received “bridge therapy” during perioperative preparation, the cessation of warfarin was too long. They believed the patient was admitted with severe symptoms of acute pancreatitis, which required urgent management and a longer cessation of warfarin would help reduce the risk of bleeding until the acute pancreatitis symptoms significantly improved. However, from our perspective as cardiologists, such a prolonged cessation inevitably increases the risk of thrombosis (16). More importantly, the combination therapy of warfarin and LMWH was not used after surgery, which inadvertently placed the patient at high risk of thrombosis. This is a consequence of insufficient clinical experience and errors in clinical decision-making. Furthermore, in many primary hospitals in our country, most patients who take oral warfarin are asked by doctors to stop taking it immediately before admission for surgery, but this may not be necessary because we do not yet know how many days of preoperative preparation the patient needs. Just like this case, the patient underwent surgery after recovering from pancreatitis, so the preoperative preparation time was slightly longer. In retrospect, it would have been possible to stop warfarin for 5–7 days after pancreatitis had improved and to simultaneously administer LMWH as “bridge therapy” until the day before surgery. Finally, the author believes that for such patients, a transthoracic echocardiography should be performed one day before or after surgery to detect valvular thrombi early and reduce the risk of stroke.

## 4 Conclusion

Patients who have undergone mechanical valve replacement surgery require lifelong anticoagulation therapy and additional surgeries can pose a risk of bleeding and thromboembolic events due to the need for perioperative anticoagulation management. It is essential to carefully manage anticoagulation therapy during and after surgery to minimize these risks. Until now, there is no consensus on the safe duration of stopping anticoagulant therapy for non-cardiac surgeries, and individualized management should be based on the patient's medical history, the type of surgery, and the risks involved. Therefore, in cases involving complex cardiac histories, interdisciplinary collaboration and consultation are extremely important. In similar cases, we should strengthen our cooperation with cardiologists to ensure that professional cardiology advice is obtained during the critical periods before, during, and after surgery.

## Data Availability

The data analyzed in this study is subject to the following licenses/restrictions: the datasets presented in this article are not readily available because of ethical/privacy restrictions. Requests to access the datasets should be directed to the corresponding authors. Requests to access these datasets should be directed to tangpli@mail3.sysu.edu.cn.
